# Advancements in cell-based therapies for the treatment of pressure injuries: A systematic review of interventional studies

**DOI:** 10.1177/20417314231201071

**Published:** 2023-11-20

**Authors:** Alianda Camesi, Reto Wettstein, Ezra Valido, Nicole Nyfeler, Stevan Stojic, Marija Glisic, Jivko Stoyanov, Alessandro Bertolo

**Affiliations:** 1 SCI Population Biobanking & Translational Research Group, Swiss Paraplegic Research, Nottwil, Switzerland; 2 Department of Plastic, Reconstructive, Aesthetic and Hand Surgery, University Hospital of Basel, Basel, Switzerland; 3 Department of Health Sciences, University of Lucerne, Lucerne, Switzerland; 4 Cardiometabolic and Respiratory Research, Swiss Paraplegic Research, Nottwil, Switzerland; 5 Institute of Social and Preventive Medicine, University of Bern, Bern, Switzerland; 6 Department of Orthopaedic Surgery, University of Bern, Bern Inselspital, Bern, Switzerland

**Keywords:** Pressure injuries, cell-based therapies, systematic review, wound healing

## Abstract

The high recurrence and complications associated with severe pressure injuries (PI) necessitate the exploration of advanced treatments, such as cell-based therapies, to facilitate wound healing. Such techniques harness the ability of different cell types to promote angiogenesis, re-epithelialization of the skin, and tissue regeneration. This systematic review explores the efficacy of cell-based therapies and tissue engineering in treating deep PI. We searched for interventional studies using cells in the treatment of PI in adults in four online libraries (PubMed, Embase, Ovid Medline, and Cochrane; latest search 10th June 2023). We found one randomized clinical trial (RCT), two non-RCT, and three pre-post studies, comprising 481 study participants with PI (253 intervention/228 controls). The risk of bias was categorized as moderate due to minimal bias in outcome measurements, or high owing to unclear patient randomization methods, as assessed by the ROBINS-I, NIH, and RoB-2 tools. Four cell types were identified in the context of cell-based therapies of PI: bone marrow mononuclear stem cells (BM-MNCs, *n* = 2); hematopoietic derived stem cells (HSC, *n* = 1); macrophages and activated macrophage suspensions (AMS, *n* = 2); and cryopreserved placental membrane containing viable cells (vCPM, *n* = 1). Wound healing outcomes were observed in patients undergoing cell-based therapies, including complete wound closure (AMS, vCPM; *n* = 142), faster healing rate (BM-MNCs, AMS; *n* = 146), improved granulation tissue formation (HSC, *n* = 3) and shorter hospitalization time (BM-MNCs; *n* = 108) compared to standard of care, with no adverse reactions. PI healing rate decreased only in one study with BM-MNC therapy, compared to control (*n* = 86). Based on the available data, though with limited evidence, it seems that macrophage deployment showed the most favorable outcomes. The results indicate that cell-based therapies offer a potential avenue for enhancing wound healing and tissue repair in PI; however, more extensive research is needed in this domain.

## Introduction

The skin, as the outermost part of the human body, serves as a protective barrier against external threats. Damage to this barrier through different means, including burns, ischemia, trauma, hypoxia, or infections can lead to two types of skin wounds: acute and chronic.^1^ Acute wounds, often result from traumatic skin damage, and heal faster than chronic wounds which persist longer than 3 months and typically are refractory to treatment. A common characteristic of chronic wounds is the persistent inflammatory state, characterized by an increased presence of neutrophils as typical biological markers.^2^ Chronic skin wounds are notably associated with peripheral arterial or venous diseases, diabetes, or prolonged immobility in patients, and impose a significant financial burden on healthcare systems.^3–5^

**Figure 1.**
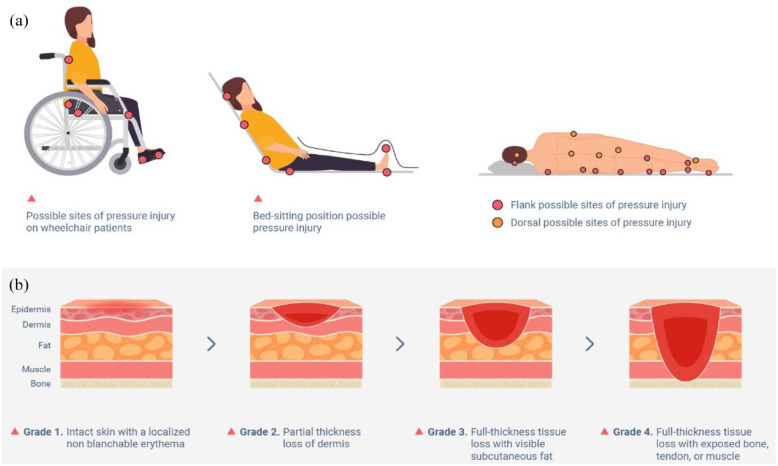
This diagram illustrates the potential sites (shown as red spots) of pressure injury occurrence in wheelchair and bed-ridden individuals ((a) possible sites of pressure injury occurrence). It also shows the various stages of PI progression from the mildest (grade I) to the most severe (grade IV) cases ((b) progression of pressure injury degeneration). In this review we included only pressure injuries from grade III to grade IV. Source: Diagram adapted from https://www.kentcht.nhs.uk/leaflet/pressure-ulcer-prevention/.

Pressure injuries (PI), also known as pressure ulcers, are a result of unrelieved pressure applied over prominent bony areas, causing a progressive ischemic tissue damage. If unaddressed, PI can lead to localized necrosis of the skin and the underlying tissues including adipose, musculotendinous, and bone tissues.^3^ PIs mainly occur over bony prominences at the heel, ankles, sides of the knees, toes and feet, and particularly in the pelvic region which includes the sacrum, coccyx (tailbone), trochanter (hip bone), and ischium (sitting erect bone) (Figure 1(a)).^6^ The National Pressure Ulcer Advisory Panel (NPUAP)^7^ has developed a widely accepted PI staging system that delineates various grades of PI degeneration (Figure 1(b)). As a rule, grade I and grade II PI can be treated nonoperatively whereas grade III and grade IV often require surgical intervention. Certain cases of deep tissue injury may improve with pressure relief alone, but most patients undergo surgical debridement and defect reconstruction. A detailed presentation of the different grades of PI and their applicable treatment is provided in Table 1.

**Table 1.** National Pressure Ulcer Advisory Panel staging system for PI.^7^

Grades	Description	Treatments
I	Intact skin with a localized non blanchable erythema; dark pigmented skin may not have visible blanching	Pressure relief, local wound care (protective dressings)
II	Partial thickness loss of dermis; a shallow open ulcer with a red-pink wound bed; may also present as an intact or ruptured serum-filled blister	Pressure relief, local wound care (moist dressing)
III	Full-thickness tissue loss with visible subcutaneous fat; bone, tendon or muscle is not exposed	*Without necrotic tissue*: Pressure relief, local wound care (moist to absorbent dressings such as hydrogel, foam, and alginate), surgical debridement, topical or systemic antibiotic *With necrotic tissue*: Sharp debridement (autolytic, enzymatic, or mechanical), moist to absorbent dressing
IV	Full-thickness tissue loss with exposed bone, tendon, or muscle; slough or eschar may be present on some parts of the wound bed	*Without necrotic tissue*: Pressure relief, local wound care (moist to absorbent dressings such as hydrogel, foam, and alginate), surgical debridement, topical or systemic antibiotic *With necrotic tissue*: Sharp debridement (autolytic, enzymatic, or mechanical), moist to absorbent dressing
Unstageable	Full-thickness tissue loss with the base of the ulcer covered by slough (yellow, tan, gray, green, or brown) or eschar (tan, brown, or black) in the wound bed	Sharp debridement to determine proper grade
Suspected deep tissue injury	Persistent non blanchable purple or maroon colored area with intact skin likely caused by shear forces; depth unknown	Pressure relief, monitor wound evaluation

PI pose substantial treatment challenges and are prone to recurrence, impacting patients’ quality of life, productivity, and life expectancy.^8^ Their problematic nature causes considerable suffering for the patient, and triggers the need for allocation of additional resources, escalating the cost burden on healthcare systems.^9^ Factors further contributing to the formation of PI include poor nutrition, inadequate sanitation, urinary and fecal incontinence, and overall diminished physical and mental health.^3^ Particularly troublesome is the fact that the most common PI, those occurring over the pelvic girdle and specifically the ischium, are hard to prevent because patients must remain seated to maintain a reasonable quality of life. Therefore, treatment and management of PI require complex interdisciplinary approach, encompassing reduction of pressure on the skin, necrotic tissue debridement, wound cleansing, managing bacterial load and colonization, and wound dressing selection.^10–12^ For deep PI (grade III and IV) surgical intervention includes fasciocutaneous and occasionally musculocutaneous flaps, skin grafts, and rarely direct closure.^13^ However, complications after PI surgery are frequent, prolonging patient isolation from social and work environments, and substantially inflating healthcare costs. Complications often include impaired wound healing, hematoma and seroma formation, and infections. Therefore, the enhancement and acceleration of wound healing has become an important focus in medical research, emphasizing the importance of the development of therapeutic treatments which promote healing processes.

Some promising techniques are being developed within the fields of regenerative medicine, gene therapy, somatic cell therapy, and tissue engineering. For example, tissue engineering aims to reconstruct the structural and functional components of the skin, reduce scar formation, and improve wound healing.^14^ Such approaches aim to accelerate healing in deep PI and aim to decrease recurrences by offering more stable results. Therefore, this systematic review intends to provide a comprehensive overview of cell-based therapies in human treatment of deep PI, identify gaps in the literature, and critically appraise the quality of the existing evidence to direct future research.

## Methods

### Data sources and search strategy

The systematic review was conducted following the recommendation of the Cochrane Methods Bias for risk of bias assessment and was reported according to the Preferred Reporting Items for Systematic Review and Meta-Analyses Protocols (PRISMA-P) guidelines (for more information on guidelines, Supplemental Material, File 1).^15^ Detailed study protocol can be found in in PROSPERO (CRD42022378212). In brief, we systematically searched four databases, namely PubMed, Embase, Ovid Medline, and Cochrane online libraries (latest search 10th June 2023; complete search strategy for Ovid Medline in Supplemental Material, File 2). We used the search terms relevant to research question, such as “stem cells,” “pressure injuries,” “cell-based therapy,” “tissue engineering” and “clinical trials.” To identify additional relevant articles, references of all included studies were hand searched, as well as studies citing included articles. We also searched in clinicaltrials.gov using the terms “Pressure Injury,” “Pressure Ulcer,” and “Cells” to identify any new clinical trials for the treatment of chronic pressure injuries.

### Study selection and eligibility criteria

Titles and abstracts were managed using the EndNote file (EndNote™20, Clarivate, v. 20.0.1). All interventional studies published in English and conducted in adult individuals (⩾18 years old) with PI in any area of the body were eligible for inclusion. Animal studies, ex vivo and in vitro studies, systematic reviews, case report, editorials, commentaries or conference abstracts were not eligible for inclusion. The full texts were examined independently by three reviewers during the initial round of the search to generate a list of relevant studies. Discrepancies in study inclusion were resolved through discussion with an experienced reviewer, who participated in the final decision.

### Data extraction and methodological quality evaluation

Data extraction was conducted by two reviewers in parallel using pre-defined extraction sheet and comprised information such as lead author’s name, publication year, study location, study design, sample/population size, patients’ demographic information (e.g. mean age), health status/comorbidities, medication use, years since PI, grades of PI, information on health outcomes and study results.

The quality of individual studies was assessed based on adequacy of sources, comparability between groups, and reliability of evidence. Each included study was evaluated using the Cochrane Methods Bias tool for assessing risk of bias. The ROBINS-I tool was used to assess the quality of non-randomized interventional studies.^16^ This quality evaluating procedure examined elements such as confounding factors in the study, selection of participants into the study, classification of intervention, deviations from intended interventions, missing data, measured outcomes, and selection of the reported results. The quality of one arm non-randomized trials was evaluated using the National Heart Lung and Blood Institute (NHLBI) Quality Assessment Tool.^17,18^ The authors conducted quality evaluations independently, followed by a consensus meeting to resolve discrepancies. To assess the quality of randomized controlled trial (RCT) studies, the RoB2 tool was used.^19^ It evaluated the quality of randomization of the studies, deviations from intended interventions, missing outcome data, outcome measurement and reported result selection. Studies were categorized into low, moderate, and high risk of bias for randomized controlled studies; and low, moderate, serious, critical, and no information for non-randomized interventional studies. We categorized studies into different levels of evidence (Level 1 to Level 4) based on the strength of their study design and the assessment of bias risk. For instance, randomized controlled trials (RCTs) with low risk of bias were classified as Level 1 evidence, while RCTs with moderate risk of bias were classified as Level 2 evidence. A detailed description of the classification process can be found in Table S5 (Supplemental Material, File 2).

## Results

### Literature search and study selection

**Figure 2.**
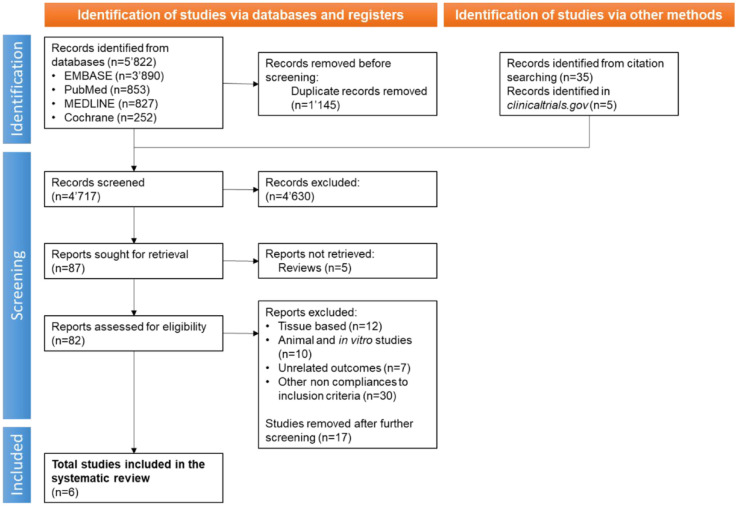
PRISMA flow diagram shows the process of inclusion and screening of studies for treatment of pressure injuries which involve cells. The flowchart illustrates the steps taken during searches of databases, screening, and reasons for exclusion.

The initial search resulted in 5822 studies, and after removing 1145 duplicates, 4677 titles and abstracts were screened (Figure 2). Through citation searching, we identified 35 additional articles and through *clinicaltrials.gov* we identified 7 clinical trials (2 duplicates), and further screened them for eligibility (for a total of 4717 titles and abstracts). Eighty-two full texts were evaluated, and ultimately six relevant articles were included in the systematic review (Table 2 and Table S1, Supplemental Material, File 2). Excluded studies were tissue-based treatments of PI, case studies or in vitro studies. Studies including different types of chronic wounds (*n* = 4), such as venous leg ulcer, diabetic foot ulcer or complex wounds, were excluded because data was not disaggregated and impossible to assign only to the PI group.^20–23^ Among the included studies, two studies featured a pre-post design where results were observed in the same patients after cell-based treatment (without control comparison). Four studies compared conventional treatment with cell-based treatment, of which three were conducted in different patients and one was conducted in the same patients across different sides of the PI. The studies involved 481 study participants, among whom 198 males, 84 females, and 199 undisclosed PI patients. Study participants were either patients with spinal cord injury or elderly people (average age ~80 years old). Controls (*n* = 228) were treated with standard of care in the same population as the cell-based therapy group.

**Table 2.** Characteristics of trials included in the systematic review.

Authors	Type of study	Total patients (gender)	Population investigated	Mean Age (yo)	Type of cell intervention	Delivery	Outcomes measured	Results		Risk of bias	Level of evidence
Burgos-Gutierrez et al., 2022	Retrospective, non-randomized controlled study	149 m;f = 128;21	SCI patients	52	BM-MNCs (*n* = 86)/ NCT (*n*-63)	Injection	Wound healing; procedure safety; treatment time	+	Accelerated rate of wound healing and wound area reduction compared to the non-stem cell therapy treatment; shorter hospitalization day in the treatment group in the first 6 months after treatment.	Moderate	Level 3
								-	1-year post-treatment, recurrence rate was significantly higher in the group treated with BM-MSC compared to the conventional surgery.		
Danon et al., 1997	Non-randomized, controlled study	199 m;f = NI	Elderly patients	81	Macrophages from blood unit (*n* = 72)/ NCT (*n* = 127)	Injection	Wound healing; procedure safety	+	Significant increase of efficacy of treatment and healing time in the treatment group; no side effects observed in the macrophage-treated group.	Moderate	Level 3
Golla and Phelan, 2019	Pre-post study	4 m;f = 2;2	SCI patients	61	vCPM	Topical	Wound closure	+	Wound closure in 7 weeks for defects repaired by vCPM and muscle flap reconstruction; Absence of complications and recurrence rates in all patients.	Moderate	Level 4
Sarasua et al., 2011	Pre-post study	22 m;f = 19;3	SCI patients	56	BM-MNCs	Injection	Clinical outcome; procedure safety; treatment time	+	Full healing observed in 19 of 22 patients; shortened of hospital stay; no ulcer recurrence in all 19 patients.	Moderate	Level 4
Wettstein et al., 2014	Pre-post study	3 m;f = 3;0	SCI patients	36	Hematopoietic derived stem cells	Injection	Cell granulation and tissue formation	+	Increased granulation tissue formation and wound contracture observed in PI side treated with stem cell.	Moderate	Level 4
Zuloff-shani et al., 2010	Prospective, randomized clinical trial	104 m;f = 46;58	Elderly patients	78	AMS (*n* = 66)/ NCT (*n* = 38)	Injection	Wound closure; time to complete wound closure; mortality	+	Increased percentage of completely closed wounds with AMS treatment.	High	Level 3
								o	No significant effect on time to complete wound closure and mortality of the patients.		

BM-MNCs: bone marrow mononuclear stem cells; vCPM: cryopreserved placental membrane containing viable cells; AMS: activated macrophage suspension; NCT: non-cell therapy.

Risk of bias evaluation for non-randomized interventional was based on ROBINS-I tool, pre-post studies on NIH tool, and for randomized controlled studies using RoB2 tool. Evaluation of level of evidence followed criteria listed in Supplemental Table S4. Results are identified as positive (+), uninfluential (o) and negative (−).

We identified four groups of cell types used across the studies, comprising: bone marrow mononuclear stem cells (BM-MNCs, *n* = 2); hematopoietic derived stem cells (HSC, *n* = 1); macrophages and activated macrophage suspension (AMS, *n* = 2); and cryopreserved placental membrane containing viable cells (cVPM, *n* = 1). Cell suspensions were either injected into the wound sites (*n* = 5) or topically applied (*n* = 1). The most common outcomes reported across the studies were complete wound closure, cell granulation and tissue formation, time to complete wound closure, procedure safety, recurrence rate and adverse events.

### Bone marrow-derived mononuclear cells

A non-randomized controlled trial study reported full healing outcomes of PI in 19 of 22 patients with spinal cord injury (SCI) patients after 19 months post-treatment. PI (grade IV) were treated with BM-MNCs and no ulcer recurrence was noted in all 19 patients.^24^ In addition, conventional surgery required 20 min of daily wound care postsurgery, while with cell-therapy treatment, only required an average of 5 min, with a reduction of mean hospital stay from 85 to 43 days. Similarly, another non-randomized controlled trial study reported a shorter hospitalization duration (length not specified in the study) when comparing BM-MNCs therapy (injection of 10 mL of cell suspension under the suture) to only direct surgical closure of PI (grade III/IV) in patients with SCI.^25^ Although the incidence of complications during the initial trimester after surgery demonstrated a comparably high percentage (>30%), the intrasurgical administration of the cell suspension negatively influenced the healing rate and led to an increased rate of recurrence. In the long-term – between 6 months and 1 year post-treatment – the recurrence rate was significantly higher (39% against 3%) in the group treated with BM-MNCs compared to the conventional surgery (fasciocutaneous flap).

### Hematopoietic derived stem cells

In a prospective pilot study, autologous hematopoietic derived stem cells (HSCs) have demonstrated a 50% reduction in the volume of PI (grade III/IV) on the treated side in comparison to a 40% reduction on the control side, providing positive influence of clinical application of HSCs on granulation tissue formation and wound contraction in SCI patients (even if not statistically significant).^26^ In the 2 years following surgery, local clinical inspections and palpation showed no signs of cancer formation.

### Placental mesenchymal stem cells on a membrane

In a retrospective non-randomized and uncontrolled study, PI (grade IV) were treated with cryopreserved placental membrane containing viable cells (vCPM), in parallel to standard musculocutaneous flap surgery. Four SCI patients showed complete wound closure in 7 weeks after treatment with vCPM, without complications and recurrence in all patients over the course of a 12-month period.^27^

### Blood-derived macrophages

In a prospective (non-parallel) controlled clinical trial, elderly patients with grade III and grade IV PI (mean age 78 years old) were treated using activated macrophage suspension (AMS) or standard of care (debridement of the wound followed by wound dressing).^28^ AMS treatment increased the percentage of completely closed wounds (69%, compared to 13% in the control group), however the cell-based treatment had no significant effect on time to complete wound closure and mortality of the patients 1 year post-treatment. Finally, in a non-RCT, elderly patients (mean age 81 years old) with PI were treated with or without local injection of macrophages, without debridement before or after the treatment with cells.^29^ In the macrophage-treated group, 27% of ulcers healed compared to only 6% of the ulcers in the control group. Neither serious negative nor negative side effects were associated with any of the leukocyte treatments.

### Quality evaluation

Quality of each study was examined using the NHLBI Quality Assessment Tool and Cochrane risk of bias tools (Tables S2–S4, Supplemental Material, File 2). Out of six studies, one was a randomized controlled trial,^28^ two of them were non-randomized controlled trials,^25,29^, three were uncontrolled pre-post studies (Table 2).^24,26,27^ Five studies demonstrated a moderate risk of bias, because the outcomes measured could be minimally influenced by knowledge of the intervention received by study participants. One study, the RCT, was classified at high risk of bias due to the unclear method of randomization of the patients included in study. Three studies (50%) were classified as Level 3,^25,28,29^, three studies were classified as Level 4 study.^24,26,27^

## Discussion

### State of art of cell-based therapies for pressure injury treatment

This systematic review examined the clinical outcomes of various cell-based therapies, compared and combined with conventional methods or standard of care, for the treatment of deep PI (grade III/IV). Cell-based therapies were used concomitantly to standard wound care, debridement, direct surgical closure or reconstructive flap surgery to support and accelerate wound healing. Overall, patients treated with cell-based therapies demonstrated an accelerated rate and increased number of wound closures, shorter hospitalization and fewer clinical complications. Specifically, the use of macrophages facilitated wound recovery in comparison to those receiving conventional treatment.

Macrophages are fundamental during wound healing stages and after injury, pro-inflammatory macrophages M1 infiltrate the wound to clear bacteria, debris and dead cells. As the tissue begins to repair, these M1 macrophages transition into anti-inflammatory macrophages M2, initiating the proliferation of fibroblast, keratinocytes, and endothelial cells into dermis, epidermis, and vasculature respectively. This process results in wound closure and formation of scar.^30^ In chronic wounds, macrophages persist in M1 pro-inflammatory type and fail to switch into M2 anti-inflammatory type, hindering the repairing of damaged tissue.^30^ Due to their fundamental roles in the wound healing stages, macrophages have already been used in clinical trials for treatment of chronic wounds. One study reported significant improvement in efficacy and healing time when using macrophages prepared from a blood unit for ulcer treatment, with no indicated side effects.^29^ Furthermore, macrophages have been noted to produce tissue debriding metalloproteinases (MMP) as well as their inhibitors, which contribute to necrotic tissue cell destruction, and cytokines to promote tissue regeneration.^31^ The use of leukocyte- and platelet-rich fibrin in the treatment of refractory leg ulcers resulted in a significant reduction in ulcer size and an increase in the rate of healing.^22^

Similar results in the treatment of PI were obtained by bone marrow mononuclear stem cells (BM-MNCs). BM-MNCs comprise a heterogeneous group of single-nucleated cells including lymphocytes, monocytes, and a small proportion of progenitor cells containing hematopoietic stem cells, mesenchymal stromal cells and endothelial progenitor cells.^32^ The mechanism of wound healing using patients’ own bone marrow relies on their abilities to promote re-epithelialization and tissue granulation.^33^ Clinically employed for the treatment of chronic ulcers, these cells are characterized by their angiogenic growth factor secretion, induction of anti-inflammatory agents, and the ability to improve neovascularization at the wound sites.^34^ The inflammation and oxidative stress generated during wound healing support a conducive environment for BM-MNCs to proliferate and self-renew and improve wound healing through differentiation and the promotion of blood vessel formation.^33^ Moreover, BM-MNCs secrete paracrine factors which recruit macrophages and endothelial cells to enhance wound healing,^35^ and they secrete fibroblast growth factor (FGF), and vascular endothelial growth factor (VEGF) which help prevent apoptosis and promote the reorganization of new matrix formation.^36^

Tissue regeneration, increased granulation tissue formation and improved reduction of wound volume was obtained by the use of hematopoietic derived stem cells (HSC) and placental membrane containing viable cells (vCPM). HSC are multipotent cells located in organs such as peripheral blood, bone marrow and umbilical cord,^37^ and they are characterized by their self-renewal and differentiation abilities to different blood cell lineages through their functional maturation process.^37^ HSCs have been used for regenerative medicine purposes and have shown potential in wound repair – with mesenchymal stromal cells – notably through the production of collagen types I and III, contributing to the long-term increased wound contraction and deposition of collagen during wound healing.^38^ Similarly, vCPM have shown to induce cell proliferation and differentiation in the tissue regenerative process.^39^ Serial vCPM applications in patients with complex wounds resulted in the development of granulation tissue, followed by wound closure.^40^ vCPM contains extracellular matrix proteins, growth factors, and viable stromal cells such as fibroblasts and stem cells which are involved in the process of maintaining the structural and cellular integrity of the tissue.^39^ Another study comparing the efficacy of a human viable wound matrix of cryopreserved placental tissue for the treatment of chronic venous leg ulcers demonstrated improved wound healing and significant reduction of ulcers in comparison to standard therapy.^41^ Their low immunogenicity to host and low invasive method to harvest, vCPM is an ideal cell product to use in the treatment of chronic wounds.

### Current hurdles of cell-based therapies and future directions

Before cell-based therapies can fully realize their potential in treating PI, several challenges must be navigated. Firstly, there’s a pressing need to diversify the cell types used in treatments. Emerging evidence, for instance, underscores the potential of mesenchymal stromal cells (MSC) in chronic wound healing, even though they haven’t been the primary focus for PI treatment in adults. Clinical trials have shown the efficacy of MSC in enhancing angiogenesis, accelerating re-epithelialization, and promoting wound closure. Beyond the promise of MSC, other cell types like keratinocytes and fibroblasts have also shown potential in treating chronic ulcers. However, the cell therapy journey isn’t without its hurdles. Ensuring the safety of these treatments is paramount, especially given concerns about immune rejection, potential tumorigenicity, and uncontrolled cell growth. Moreover, the need for refining and optimizing clinical trial protocols to ensure consistent and reliable outcomes is evident. These challenges, along with their intricate details, are reviewed in depth in the subsequent sections.

### Alternative cell types for treatment of PI

To enhance the efficacy of cell therapies for the treatment of PI, we recommend to wider the range to other cell types. For instance, although no studies have exclusively used mesenchymal stromal cells (MSC) only for PI treatment in adults, emerging evidence suggests their potential. MSC treatment for chronic wound healing enhances angiogenesis, accelerates re-epithelialization, improves granulation, and speeds up wound closure.^5^ Some clinical trials using MSC for the treatment of chronic wounds have also been reported. For instance, autologous bone marrow mesenchymal stem cells (BM-MSC) used on diabetic foot ulcers improved pain-free walking and reduced ulcer size by 72%.^42^ Wound closure was observed in 8 weeks after topical application of cultured and profiled BM-MSC in chronic lower limb patients followed by faster healing rate with increased cell application.^43^ Another study described the use of MSC on collagen sponges used as wound dressings on intractable dermatopathies patients, in which 18 out of 20 patients reached complete wound healing.^44^ The safety and efficacy of human BM-MSC has been proven by utilizing immunodeficient murine strains such as non-obese diabetic mice.^45^

Other clinical trials applying umbilical cord stem cells, fibroblasts, and adipose-derived MSC have also been used in the treatment of chronic wounds mainly in critical limb ischemia, diabetic, and venous leg ulcer patients.^46^ Keratinocytes – which constitute about 90% of epidermal cells and play key role in skin repair – have been widely used in the treatment of chronic ulcers.^20,46^ They can be administered as cryopreserved cultured allografts or suspended with dermal cells to improve chronic wound healing. Allogeneic cultured-keratinocyte-collagen dressing stimulated wound healing by growth factors and cytokines released in situ, reducing pain in 80% of cases and promoting granulation tissue in the ulcer bed in 70% of cases.^20^ Keratinocytes cultured on a thick collagen gel with the addition of fibroblasts demonstrated a well differentiated epidermis with stratum corneum and stratum granulosum, suggesting that autologous skin cell suspension exert substantial advantages for the treatment of chronic wounds.^21,47^ Indeed, Kuroyanagi et al. showed that fibroblasts had a good biocompatibility also with the PI and the process of wound healing was significantly accelerated in comparison to the control group, with a greater rate of epithelialization.^23^ Moreover, no adverse reactions, such as infection and inflammation, were observed. These findings suggest that different types of cells could potentially be used as alternative cell therapy for the treatment of PI.

### Challenges of cell-based therapies

This systematic review found no adverse events related to the treatment of PI with cells after 6 months to 2 years monitoring period. PI recurrence was not reported in almost all patients after intervention with cells, however there was an increased relapse in BM-MNC-based therapy after 1 year.^25^ Whether this relapse highlights a potential downside in the use of BM-MNC is unclear because the initial healing rate within the initial 3 months following surgical closure remained indistinguishable, regardless of the cell therapy. The claimed shorter hospitalization due to accelerated wound healing was not confirmed by the presented data. The authors suggest that differences in bacterial contamination could have led to a higher relapse rate in the group treated with BM-MNC. Nevertheless, it is important to consider that recurrence of PI is multifactorial and mainly depends on the local amount of high pressure for a prolonged period of time, so the influence of BM-MNC-based therapy should not be overstated.

Safety and efficacy of cell-based therapies depend on several factors including the risk of immune rejection, tumorigenicity and uncontrolled cell growth, manufacturing process, and regulatory challenges. Introducing cells from external sources into a patient’s body might trigger an immune response and result in the rejection of transplanted cells, especially when conducting allogenic transplantations.^48^ In this systematic review, we included studies utilizing patient’s own cells for the treatment of PI, showing no immunogenicity reaction in all patients, but it is crucial to take in account the compatibility of the cells being applied. Additionally, when selecting the appropriate cell type to use in cell-based therapies, it is critical to consider the risk of cells undergoing malignant transformation, or to support tumor growth within the recipient’s body. For example, the use of keratinocytes stem cells (KSC) has raised concerns due to their observed resistance to apoptosis, indicating a potential association with the initiation and progression of skin cancer.^46^ It is therefore advisable to closely monitor patients long-term for any signs of tumor formation when considering the use of KSC as a treatment for PI. Furthermore, the understanding of the long-term effects of cell-based therapies remains incomplete. Some patients may experience delayed adverse reactions to cell-based therapies several years after treatment, while others may remain unaffected, highlighting the importance of gaining a better understanding of these therapies in order to enhance both their safety and efficacy.^49^ For example, undisclosed rate of apoptotic cells present in the cell-based product or unsure homing capacity of cells applied topically, rise concerns regarding their impact on the patient’s well-being.^50,51^

Ensuring the safety of cell-based therapies is of main importance, necessitating stringent quality control measures during the cell harvesting process to prevent any potential contamination by bacteria, viruses, or other pathogens that may pose harm to patients. In the clinical practice, surgical interventions would benefit from utilizing a closed system instrument specifically engineered for the extraction of cells from the own patient’s tissue. The adoption of such a method would not only reduce potential contaminations but would also help to ease the regulatory challenges associated with cell-based therapies. The development of cell-based therapies as alternative treatments necessitate adherence to stringent regulatory requirements, and dealing with the regulatory process can be both time-consuming and expensive. The limited availability of research projects focusing on cell-based therapies for the treatment of PI can be also attributed to the high cost associated with this treatment, thereby restricting access for patients within certain healthcare systems or regions. For example, production of autologous MSC require in vitro culturing, which introduces contamination risks, treatment delays, and increased costs. Instead, macrophages and BM-MNC can be obtained in abundant quantities without extensive manipulation and can be transplanted directly, in a closed system, without the need for in vitro expansion. Consequently, it is crucial to demonstrate the economic and practical benefits of cell-based therapies in comparison to traditional treatments.

### Future clinical trials

Despite the encouraging preliminary findings, further high quality, larger-scale randomized controlled trials are crucial to conclusively validate the safety, efficacy, and cost-effectiveness of cell-based therapy in managing PI. Our findings suggest that future clinical trials need the inclusion of statistically relevant numbers of patients and the appropriate control group to compare conventional therapies with cell-based therapies. During the process of patient selection, characteristics like sex and age of the patients, as well as the etiology, location, and size of PI must be similar between the groups treated either with conventional therapy/surgery only or in combination with cell-based therapies. Furthermore, double-blinded trials are essential to ensure transparency of data obtained from the results, reduce bias, and obtain a higher level of evidence. Finally, cell-based therapies for treating PI should adhere to robust protocols that clearly outline the specific cell types and tissue sources used, the required dosage for effective treatment, recommended frequency of cell application for different grades of PI, and the appropriate methods for assessing treatment outcomes.

The patient demographic mostly affected by PI typically includes the elderly, individuals with spinal cord injuries, and intensive care unit patients. Hence, it is crucial to consider the potential limitations, that is, copresence of different diseases, that may affect future clinical trials.

For example, autologous MSC isolated from diabetic individuals, when examined for their phenotype and function, proved to be less effective, exhibiting a reduced expression of VEGF and chemokine receptor CXCR4.^52^ Or, elderly patients with PI often have inadequate nutritional status which limits the withdrawal of the required volumes of bone marrow, potentially worsening their overall health condition. However, Yoshikawa et al. reported that the cell-based treatment of dermatopathies with BM-MSC activated healing mechanisms and therapeutic effects, irrespective of the patient’s age.^44^ On the other hand, future trails must consider the issue of cellular senescence when considering stem cell-based treatments for pressure injuries in elderly individuals.^53^ Cellular senescence is a state in which the cells lose their ability to divide and function effectively, a phenomenon that increases with age. This process can decrease the effectiveness of cell-based treatments, as the proliferative and differentiation capacities of autologous cells are significantly reduced.^54^

Another methodological consideration for future cell-based trials is the refinement of cell delivery. In the case of superficial ulcers, cells can be readily injected into the wound or applied topically, however in long-standing ulcers with a thick layer of dense scar tissue, injecting cells can be hard to perform and may be accompanied by cases of calcifications.^55^ Improvements to injection can be achieved by the use of supportive scaffolds, such as fibrin glue^43^ or collagen matrices.^56^ An optimal scaffold intended for wound management should retain specific characteristics, including biocompatibility, biodegradability, bioactivity, predisposition for cellular adhesion and infiltration, minimal toxicity, and minimal inflammatory response.^57^ Specifically for PI, it is essential that scaffolds demonstrate resilience to applied force, shear stress and friction.^58^

### Limitations of the study

To our knowledge, this is the most comprehensive overview of treatment of PI with cell-based approaches. However, this systematic review has some limitations that are inherent to the underlying evidence. First, the studies analyzed in this systematic review predominantly had a low level of evidence (50% at level 3 and 50% at level 4), mostly due to biased assessment of outcomes associated with PI. Second, due to the limited number of eligible studies (*n* = 6), this review included one tissue-based therapy studies (vCPM) because we considered the membrane just as a cellular support for placental MSC.

## Conclusion

**Figure 3.**
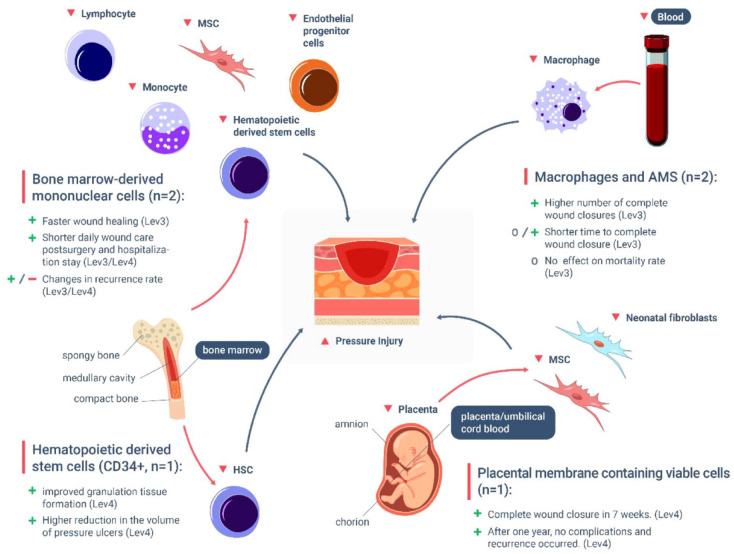
Diagram summarizing the various cell types that have been used in therapeutic approaches to treat deep pressure injuries (PI). These include bone marrow-derived mononuclear cells (BM-MNCs), hematopoietic derived stem cells (HSCs), placental membrane containing viable cells (vCPM) and macrophages and activated macrophages suspension (AMS). Research has shown that when patients undergo cell-based therapies utilizing these cell types, positive results are observed in regards to number of complete wound closures, faster healing rate, shorter hospitalization, and in most of the case no reported recurrence of the injuries. Only one study reported increased PI recurrence with BM-MNC treatment after a period of 1 year. Notably, cells isolated from the bone marrow and placenta have resulted in encouraging structural improvements to the degenerated tissue, whereas macrophages have been effective in modulating the underlying inflammatory state of pressure injuries. (Outcomes are identified as positive (+), uninfluential (o) and negative (−)).

In general, PI have low healing rates due to underlying characteristics such as excessive bacterial colonization, diminished capillary perfusion, local tissue hypoxia and altered cellular and systemic stress responses, rendering them recalcitrant to standard care treatment.^59^ Additionally, PI remain a considerable burden for people with limited mobility and for the healthcare system. This systematic review summarizes how cell-based therapies can improve PI treatment and healing (Figure 3). Stem and other cell types contribute to wound healing promoting host immune response and decreasing local inflammation. They also promote vascularization and angiogenesis by secreting growth factors and cytokines to induce cell proliferation. Furthermore, stem cells can differentiate into fibroblast, epithelial, and endothelial cells to contribute to re-epithelialization and functional regeneration of the skin structural layers. Finally, stem cells stimulate tissue regeneration, wound repair, and increase tensile strength of the skin. Clinical trials have also demonstrated the safety, appropriateness, and suitability to use cell therapies with significant potential for closing PI without adverse events. However, this systematic review highlights the need for further, well-designed research into cell-based therapies for PI treatment. The potential for using other cell types such as MSC is emerging, and keratinocytes and fibroblasts have also been successfully used in the treatment of other chronic ulcers, suggesting it may be beneficial to use a broader range of cell types to treat PI. In conclusion, cell-based therapies should be considered as alternatives or adjuvants for recalcitrant PI wound treatment, and especially in consideration of the promising results obtained with macrophage suspension.

## Supplemental Material

sj-docx-1-tej-10.1177_20417314231201071 – Supplemental material for Advancements in cell-based therapies for the treatment of pressure injuries: A systematic review of interventional studies Click here for additional data file. Supplemental material, sj-docx-1-tej-10.1177_20417314231201071 for Advancements in cell-based therapies for the treatment of pressure injuries: A systematic review of interventional studies by Alianda Camesi, Reto Wettstein, Ezra Valido, Nicole Nyfeler, Stevan Stojic, Marija Glisic, Jivko Stoyanov and Alessandro Bertolo in Journal of Tissue Engineering

sj-docx-2-tej-10.1177_20417314231201071 – Supplemental material for Advancements in cell-based therapies for the treatment of pressure injuries: A systematic review of interventional studies Click here for additional data file. Supplemental material, sj-docx-2-tej-10.1177_20417314231201071 for Advancements in cell-based therapies for the treatment of pressure injuries: A systematic review of interventional studies by Alianda Camesi, Reto Wettstein, Ezra Valido, Nicole Nyfeler, Stevan Stojic, Marija Glisic, Jivko Stoyanov and Alessandro Bertolo in Journal of Tissue Engineering
