# Supplementary material for: Advancements in cell-based therapies for the treatment of pressure injuries: A systematic review of interventional studies
Source: J Tissue Eng. 2023 Nov 20;14:20417314231201071. doi: 10.1177/20417314231201071 (PMC10658773; doi:10.1177/20417314231201071)
Supplement: sj-docx-2-tej-10.1177_20417314231201071 – Supplemental material for Advancements in cell-based therapies for the treatment of pressure injuries: A systematic review of interventional studies [file sj-docx-2-tej-10.1177_20417314231201071.docx]

**Advancements in cell-based therapies and tissue engineering for the treatment of pressure injuries: a systematic review of interventional studies.**

**Supplemental material, file 2**

Search strategy for Ovid Medline online database (827 hits):

"exp Pressure Ulcer/ OR (bedsore* OR ((bed OR decubitus OR pressure OR decubitus) ADJ3 (sore* OR ulcer*)).ti,ab.) AND (exp Tissue Engineering/ OR (tissue ADJ3 engineering) OR exp Cell- and Tissue-Based Therapy/ OR ((cell OR tissue-based OR tissue based) ADJ3 therapy) OR exp Autografts/OR autograft* OR autotransplant* OR (autologous ADJ3 transplant*) OR exp Stem Cell Transplantation/OR ((stem cell) ADJ3 transplant*) OR exp Biological Dressings/OR ((allograft OR amniotic membrane OR biologic* OR heterograft OR homograft OR pig skin OR procrine xenograft OR xenograft) ADJ3 dressing*) OR exp Stem Cells/OR ((colony forming OR colony-forming OR mother OR progenitor* OR stem) ADJ3 (unit* OR cell*)) OR exp Leukocytes, Mononuclear/OR ((mononuclear OR pbmc peripheral blood mononuclear OR peripheral blood human mononuclear) ADJ3 (leukocyte* OR cell*)) OR exp Stromal Vascular Fraction/OR ((adipose OR adipose stromal vascular OR autologous adipose OR autologous adipose stromal vascular OR stromal vascular) ADJ3 (svf OR fraction*)) OR exp Regenerative Medicine/OR (regenerative ADJ3 medicine*) OR exp Reconstructive Surgical Procedures/OR ((cosmetic reconstructive surgical) ADJ3 (surger* OR procedure*)).ti,ab.) NOT (exp Rats/OR Rat OR Rattus OR ((norvegicus OR laboratory) ADJ3 rat*) OR exp Mice/OR Mus OR Mouse OR ((mus OR mouse OR mice) ADJ3 (domesticus OR musculus OR house OR Swiss OR laboratory)) OR exp Swine/OR Suidae OR Pig* OR Warthog* OR Phacochoerus OR (wart ADJ3 hog).ti,ab.)"

**Keywords used in the search.**

- Pressure Ulcer
- Tissue Engineering
- Cell –and Tissue- Based Therapy
- Autografts
- Stem Cell Transplantation
- Biological Dressings
- Stem Cells
- Leukocytes, Mononuclear
- Stromal Vascular Fraction
- Regenerative Medicine
- Reconstructive Surgical Procedures

**Table S1.** Characteristics of studies included in the systematic review (n=6)

| **Characteristics** | **No. of studies** | **Citations** |
| --- | --- | --- |
| Level of injury |  |  |
| Tetraplegia | 0 |  |
| Paraplegia | 1 | Golla & Phelan, 2019 |
| Mixed | 2 | Burgos *et al.* 2021; Wettstein *et al.* 2019 |
| Not reported | 3 | Danon *et al.* 1997; Sarasua *et al.* 2011; Zuloff *et al.* 2010 |
| Proportion of complete injury |  |  |
| 100% | 0 |  |
| Mixed | 1 | Burgos *et al.* 2021 |
| Not reported | 5 | Danon *et al.* 1997; Golla & Phelan, 2019; Sarasua *et al.* 2011; Wettstein *et al.* 2019 ; Zuloff *et al.* 2010 |
| Duration of injury (years), range |  |  |
| ≤ 1-10 | 1 | Golla & Phelan, 2019 |
| >10 | 1 | Burgos *et al.* 2021 |
| Not reported | 4 | Danon *et al.* 1997; Sarasua *et al.* 2011; Wettstein *et al.* 2019 ; Zuloff *et al.* 2010 |
| Sex |  |  |
| Male only | 1 | Wettstein *et al.* 2014 |
| Both | 5 | Burgos *et al.* 2021; Danon *et al.* 1997; Golla & Phelan, 2019; Sarasua *et al.* 2011; Zuloff *et al.* 2010 |
| Female only | 0 |  |
| Not reported | 0 |  |
| Study size |  |  |
| <50 | 3 | Golla & Phelan, 2019; Sarasua *et al.* 2021; Wettstein *et al.* 2014 |
| 50-100 | 0 |  |
| >100 | 3 | Burgos *et al.* 2021 ; Danon *et al.* 1997 ; Zuloff *et al.* 2010 |
| Not reported | 0 |  |
| Age, (Mean) |  |  |
| 16-40 | 1 | Wettstein *et al.* 2014 |
| >40-65 | 3 | Burgos *et al.* 2021; Golla & Phelan, 2019; Sarasua *et al.* 2011 |
| >65 | 2 | Danon *et al.* 1997; Zuloff *et al.* 2010 |
| Not reported | 0 |  |
| Location |  |  |
| Europe | 3 | Burgos *et al.* 2021; Sarasua *et al.* 2011; Wettstein *et al.* 2014 |
| North America | 1 | Golla & Phelan, 2019 |
| South America | 0 |  |
| Asia | 2 | Danon *et al.* 1997; Zuloff *et al.* 2010 |
| Africa | 0 |  |

**Table S2.** Risk of bias evaluation for non-randomized interventional studies using ROBINS-I tool.

| **Study** | **Bias 1** | **Bias 2** | **Bias 3** | **Bias 4** | **Bias 5** | **Bias 6** | **Bias 7** | **Overall Bias** |
| --- | --- | --- | --- | --- | --- | --- | --- | --- |
| Burgos *et al.*,2021 | Low | Low | Low | Low | Low | Moderate | Low | Moderate |
| Danon *et al.*, 1997 | Low | Low | Low | Low | Low | Moderate | Low | Moderate |

Bias 1 (bias due to confounding); Bias 2 (bias to selection of participants into the study); Bias 3 (bias classification of interventions); Bias 4 (Bias due to deviations from intended interventions); Bias 5 (bias due to missing data); Bias 6 (Bias in measured outcome); Bias 7 (bias in selection of the reported result).

**Table S3.** Risk of bias evaluation for randomized controlled studies using RoB2 tool.

| **Study** | **Bias 1** | **Bias 2** | **Bias 3** | **Bias 4** | **Bias 5** | **Overall Bias** |
| --- | --- | --- | --- | --- | --- | --- |
| Zuloff-shani *et al.*, 2010 | High | Low | Low | Moderate | Low | High |

Bias 1 (randomization); Bias 2 (deviation from intended intervention); Bias 3 (missing outcome data); Bias 4 (measurement of the outcome); Bias 5 (selection of the result).

**Table S4.** Risk of bias evaluation for pre-post studies using the NIH tool.

| **Study** | **1** | **2** | **3** | **4** | **5** | **6** | **7** | **8** | **9** | **10** | **11** | **12** | **Risk of Bias^1^** |
| --- | --- | --- | --- | --- | --- | --- | --- | --- | --- | --- | --- | --- | --- |
| Golla & Phelan, 2019 | Y | N | Y | Y | N | Y | Y | N | Y | NR | N | NA | Moderate (6/12, 50%) |
| Sarasua *et al.*, 2011 | Y | N | Y | Y | N | Y | Y | N | Y | NR | Y | NA | Moderate (7/12, 58%) |
| Wettstein *et al.*, 2014 | Y | N | Y | Y | N | Y | Y | N | Y | NR | N | NA | Moderate (6/12, 50%) |

Abbreviations: Y, yes; N, No; NA, not applicable; NR, not reported

^1^Risk of bias rating = Low (75-100%), Moderate (50-75%), or High (0-49%)

Criteria used to assess risk of bias of pre-post study without control group:

1. Was the study question or objective clearly stated?
2. Were eligibility/selection criteria for the study population prespecified and clearly described?
3. Were the participants in the study representative of those who would be eligible for the test/service/intervention in the general or clinical population of interest?
4. Were all eligible participants that met the prespecified entry criteria enrolled?
5. Was the sample size sufficiently large to provide confidence in the findings?
6. Was the test/service/intervention clearly described and delivered consistently across the study population?
7. Were the outcome measures prespecified, clearly defined, valid, reliable, and assessed consistently across all study participants?
8. Were the people assessing the outcomes blinded to the participants' exposures/interventions?
9. Was the loss to follow-up after baseline 20% or less? Were those lost to follow-up accounted for in the analysis?
10. Did the statistical methods examine changes in outcome measures from before to after the intervention? Were statistical tests done that provided p values for the pre-to-post changes?
11. Were outcome measures of interest taken multiple times before the intervention and multiple times after the intervention (i.e., did they use an interrupted time-series design)?
12. If the intervention was conducted at a group level (e.g., a whole hospital, a community, etc.) did the statistical analysis take into account the use of individual-level data to determine effects at the group level?

| **Table S5.** Eligible study designs and criteria for classifying the level of evidence for individual studies. | | | |
| --- | --- | --- | --- |
| **Level of evidence** | **Study design** | **Study quality score based on NIH** | **Description study design** |
| Level 1 study | Randomized controlled trial | Low risk of bias | Using within-subjects comparison with randomized conditions or cross-over designs |
| Level 2 study | Randomized controlled trial | Moderate risk of bias | Using within-subjects comparison with randomized conditions or cross-over designs |
|  | Non-randomized controlled trial | Low risk of bias | Comparing intervention vs. control groups (not randomly allocated) |
|  | Prospective cohort study | Low risk of bias | Longitudinally comparing at least two similar groups (one exposed one unexposed) |
|  | Case-control study | Low risk of bias | Using a case-control study design to compare two types of diet/dietary patterns |
| Level 3 study | Randomized controlled trial | High risk of bias | Using within-subjects comparison with randomized conditions or cross-over designs |
|  | Non-randomized controlled trial | Moderate risk of bias | Comparing intervention vs. control groups (not randomly allocated) |
|  | Prospective cohort study | Moderate risk of bias | Longitudinally comparing at least two similar groups (one exposed one unexposed) |
|  | Retrospective cohort study | Low/moderate risk of bias | Retrospectively comparing an exposed/interventional group to a historical control group |
|  | Case-control study | Moderate risk of bias | Using a case-control study design to compare two types of diet/dietary patterns |
|  | Pre-post study | Low risk of bias | Using a baseline measure, intervention and a post-test in a single group |
| Level 4 study | Non-randomized controlled trial | High risk of bias | Comparing intervention vs. control groups (not randomly allocated) |
|  | Prospective cohort study | High risk of bias | Longitudinally comparing at least two similar groups (one exposed one unexposed) |
|  | Retrospective cohort study | High risk of bias | Retrospectively comparing an exposed/interventional group to a historical control group |
|  | Pre-post study | Moderate or high risk of bias | Using a baseline measure, intervention and a post-test in a single group |
|  | Cross-sectional study | Regardless of the quality score | Comparing two groups exposed and unexposed to specific diet/micro- or macronutrient |
